# Accessing the potential impact of education about cancer screening programs: a pilot before and after study

**DOI:** 10.1080/07853890.2021.1896842

**Published:** 2021-09-28

**Authors:** Maria Inês Teodoro, Hugo Torres, Nuno Venâncio, Filipa Alves da Costa

**Affiliations:** a Instituto Universitário Egas Moniz (IUEM), Egas Moniz Cooperativa de Ensino Superior, Caparica, Portugal; b Centro de Investigação Interdisciplinar Egas Moniz (CiiEM), Egas Moniz Cooperativa de Ensino Superior, Caparica, Portugal

## Abstract

**Introduction:**

In the context of the Public Health curricular unit, students were requested to choose a relevant theme within the scope of public health and to develop a field study involving the community and ideally draw an intervention impacting on their well-being. We identified breast, cervical and colorectal cancer as a priority [1]. Our aim was to evaluate the citizens’ knowledge about screening and then subsequently to raise their awareness of the importance of screening. Our ultimate goal was to increase knowledge by providing written information on the theme.

**Materials and methods:**

We conducted a pilot before and after study aiming to involve 10% of the estimated sample (700 adult individuals; for an 11% increase [2], 5% Type I and 20% type II errors using *Sample Size Calculator*) living in the municipalities of Almada, Santarém and Lisboa. To evaluate their knowledge, we developed a 20-item questionnaire containing statements to be rated using a 4 point Likert scale, scored for a maximum of 80 points. We assessed knowledge at baseline and 48 h following the intervention. The intervention consisted of the delivery of a flyer containing general information on cervical cancer, breast cancer and colorectal cancer prevention and early detection means. Data analysis resorted to paired samples *t*-test using IBM SPSS Statistics, version 25.0, considering a 95% confidence interval.

**Results:**

The sample included 70 people aged between 23 and 70 years, 73% female, with 56% having university education. Most respondents considered important to engage in screening programs, at both time points (70%); however, only 56% knew where these could be sought, as opposed to 68% following intervention. Mean baseline knowledge was 63.27 points, increasing to 73.54 post intervention, i.e. 10.27 points corresponding to a significant increase of 12.37% (*p-*value ≤ .05).

**Discussion and conclusions:**

Our data suggests that through a very simple educational intervention it is possible to improve the population’s knowledge about cancer screening programs, with impacts exceeding previously reported studies [2]. We should acknowledge our sample is limited (10%) and probably biased as educational levels reported were quite high. This apparent limitation, however, suggests we may even produce greater differences targeting our intervention at lower literate individuals. Future studies should focus on the link between knowledge and behaviour as the literature suggests intention may have a role in mediating this association [3].
